# Artificial Lung Gas Exchanges Depend On Ecmo Settings

**DOI:** 10.1186/2197-425X-3-S1-A514

**Published:** 2015-10-01

**Authors:** F Mojoli, S Bianzina, I Bianchi, G Tavazzi, S Mongodi, M Pozzi, A Orlando, A Braschi

**Affiliations:** Anesthesia and Intensive Care, Fondazione IRCCS Policlinico S. Matteo, University of Pavia, Pavia, Italy

## Introduction

Artificial membrane lung (AL) gas exchanges are usually evaluated according to PaO_2_/FiO_2_ ratio. in addition, dead space ventilation and shunt fraction can be measured by the same equations used for native lungs [1].

## Objectives

To study the effect of AL settings - gas flow (GF), blood flow (BF) and FiO_2_ - on AL PaO_2_/FiO_2_ ratio, dead space and shunt, to suggest how to properly monitor these parameters.

## Methods

We performed three different tests:

a) GF changes (from 1 to 10 L/min) in 8 AL at clinically set BF and FiO_2_;

b) BF changes in 6 AL at constant FiO_2_ (1) and GF (10 L/min);

c) FiO_2_ = 1 vs. clinically set FiO_2_ in 10 AL at clinically set BF and GF.

We performed pre- and post-oxygenator blood gas analysis and measured CO_2_ at AL exhaust port by sidestream capnography, in order to evaluate PaO_2_/FiO_2_ ratio, dead space and shunt.

## Results

**Figure 1:**
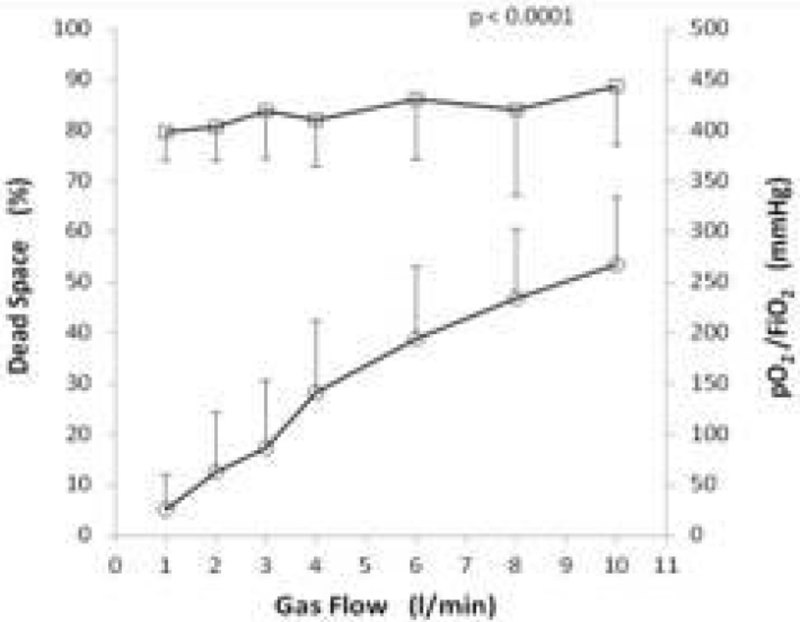
Figure 1

**Figure 2:**
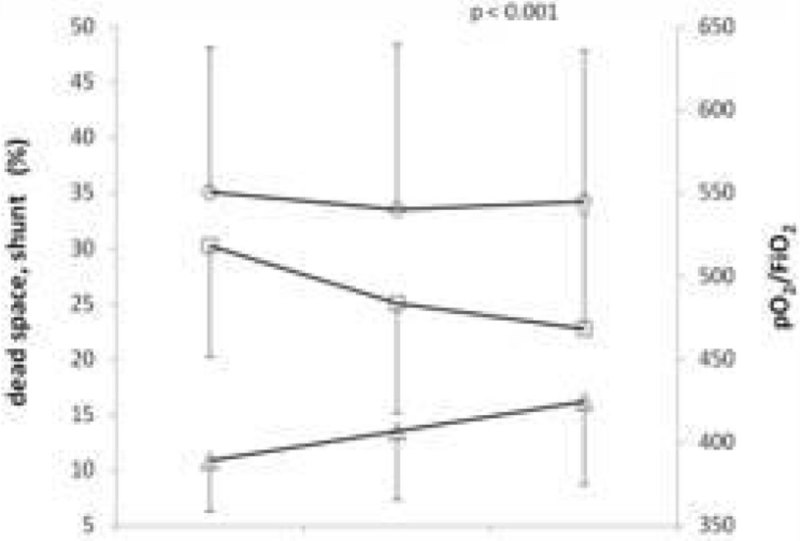
Figure 2

a) At clinically set BF (4.0 ± 0.9 L/min) and FiO_2_ (0.87 ± 0.15), PaO_2_/FiO_2_ ratio did not correlate with GF, whereas dead space progressively increased with GF (R = 0.7904, p < 0.0001) (Figure 1).

b) Data were collected at basal (3.3 ± 0.7 L/min), increased (4.1 ± 0.8 L/min) and decreased (2.5 ± 0.6 L/min) BF. With the progressive increase of BF, dead space did not change, whereas PaO_2_/FiO_2_ ratio decreased and shunt increased (p < 0.001) (Figure 2).

c) At clinically set BF (3.5 ± 1.1 L/min) and GF (5.1 ± 2.4 L/min), the mean difference ± standard deviation of PaO_2_/FiO_2_ ratio and shunt obtained at clinically set (0.80 ± 0.20) vs. FiO_2_ = 1 was -76 ± 109 mmHg and 2.1 ± 11.6%, respectively.

## Conclusions

To properly monitor AL CO_2_ removal and oxygen transfer, evaluations should be performed at constant GF and at FiO_2_ = 1 and constant BF, respectively.

## References

[1] Castagna L, Zanella A, Scaravilli V, Magni F, Deab SA, Introna M (2015). Effects on membrane lung gas exchange of an intermittent high gas flow recruitment maneuver: preliminary data in veno-venous ECMO patients. J Artif Organs.

